# P-354. Clinical Utility of Tuberculosis Screening Tests After Tuberculosis Exposure at an Infusion Clinic

**DOI:** 10.1093/ofid/ofae631.555

**Published:** 2025-01-29

**Authors:** Aldon Li, Tawny Belleau, Graciela Faiad

**Affiliations:** Kaiser Permanente; Kaiser Permanente; Kaiser Permanente

## Abstract

**Background:**

Tuberculin skin testing (PPD) and interferon gamma release assays (IGRA) facilitate screening of patients exposed to active tuberculosis (TB), but the clinical utility of TB screening tests in determining management of exposed high-risk patients is not well described. Because the sensitivity of TB screening tests is dependent on patient’s immunocompetency, further studies looking at the correlation between exposure, testing, and risk of progression to active TB in immunocompromised patients are needed. This study aims to describe TB testing and TB transmission among a large cohort of immunocompromised patients after a known TB exposure event.

**Methods:**

Retrospective case series of patients who were exposed to TB as defined by CDC Guidelines, identified through contact tracing reports during exposure period April-July 2019. Local infection control protocol was to assess exposed patients with an IGRA initially, then place a PPD if IGRA was inconclusive.

**Results:**

During the exposure period, an employee with direct patient care duties at an oncology infusion clinic developed active TB, exposing 443 patients. Of the 443 exposed patients, 57 either passed away prior to testing or declined testing. Of the 386 patients who underwent screening tests, 351 (91%), 23 (6%), and 12 (3%) were IGRA negative, positive, and indeterminate, respectively. Of the 12 IGRA indeterminate patients, 8 (67%) had a negative PPD, 2 (16%) refused PPD testing and a repeat IGRA turned negative, and 2 (16%) passed away without further testing. Of the 23 IGRA positive patients, latent TB infection (LTBI) treatment was given in 16 (70%) patients with 7 patients either declining therapy or passed away. None of the 443 patients developed active TB after a 5 year follow up.

**Conclusion:**

The results of this study found no TB transmission occurred during a high-risk exposure event involving immunocompromised patients when protocol driven testing, treatment, and follow-up was completed. Although CDC publications report low sensitivities of IGRAs in immunocompromised patients, given the large number of exposed patients, serum testing was a practical choice for initial exposure testing and this study showed successful interventions when paired IGRA-PPD testing was used to risk stratify high-risk patients for LTBI therapy.

**Disclosures:**

**All Authors**: No reported disclosures

